# Safety analysis and complications of condylar screws in a single-surgeon series of 250 occipitocervical fusions

**DOI:** 10.1007/s00701-021-05039-z

**Published:** 2021-11-25

**Authors:** Sharon Ka Po. Tam, Paolo A. Bolognese, Roger W. Kula, Andrew Brodbelt, Mansoor Foroughi, Marat Avshalumov, Denmark Mugutso, Ilene Ruhoy

**Affiliations:** 1 Royal Sussex County Hospital, Brighton and Sussex University Hospitals NHS Trust, Brighton, UK; 2 Division of Neurosurgery, Chiari/EDS Neurosurgical Center, Mount Sinai South Nassau, Oceanside, NY USA; 3 Division of Neurosurgery, The Walton Centre NHS Foundation Trust, Liverpool, UK; 4 Neurological Surgery Professional Corporation, Rockville Centre, NY USA; 5 Division of Neurology, Chiari/EDS Neurosurgical Center, Mount Sinai South Nassau, Oceanside, NY USA

**Keywords:** Occipitocervical fusion, Occipital condyle screw, Occipital condyle, Cranial vertebral fixation

## Abstract

**Objective:**

Condylar screw fixation is a rescue technique and an alternative to the conventional configuration of occipitocervical fusion. Condylar screws are utilized when previous surgical bone removal along the supraocciput has occurred which makes anchoring of a traditional barplate technically difficult or impossible. However, the challenging dissection of C0-1 necessary for condylar screw fixation and the concerns about possible complications have, thus far, prevented the acquisition of large surgical series utilizing occipital condylar screws. In the largest case series to date, this paper aims to evaluate the safety profile and complications of condylar screw fixation for occipitocervical fusion.

**Methods:**

A retrospective safety and complication-based analysis of occipitocervical fusion via condylar screws fixation was performed.

**Results:**

A total of 250 patients underwent occipitocervical fusions using 500 condylar screws between September 2012 and September 2018. No condylar screw pullouts, or vertebral artery impingements were observed in this series. The sacrifice of condylar veins during the dissection at C0-1 did not cause any venous stroke. Hypotrophic condyles were found in 36.4% (91 of the 250) cases and did not prevent the insertion of condylar screws. Two transient hypoglossal deficits occurred at the beginning of this surgical series and were followed by recovery a few months later. Corrective strategies were effective in preventing further hypoglossal injuries.

**Conclusions:**

This surgical series suggests that the use of condylar screws fixation is a relatively safe and reliable option for OC fusion in both adult and pediatric patients. Methodical dissection of anatomical landmarks, intraoperative imaging, and neurophysiologic monitoring allowed the safe execution of the largest series of condylar screws reported to date. Separate contributions will follow in the future to provide details about the long-term clinical outcome of this series.

## Introduction

Occipito-cervical (OC) fusions are used to treat a variety of pathologies affecting the craniocervical junction [2, 5, 7, 8, 20].

Condylar screws have been recently introduced as a rescue technique for OC fusions, whenever an existing craniectomy, or a fractured or thin supraocciput limit the available fixation points to the occipital squama [1, 21]. Concerns regarding potential complications have slowed the use of this technique thus far. In this study, we present the complications and safety analysis of a single-surgeon series of 250 occipitocervical fusions (500 condylar screws).

## Methods

### Inclusion criteria

Clinical criteria: (1) Karnofsky score ≤ 70, after failure of conservative management, and (2) 75% improvement of chief complaints from the baseline, during invasive cervical traction.

Plus one or more of the following radiological criteria: (1) basilar impression or basilar invagination; (2) clivo-axial angle (CXA) of 135° or below, on neutral imaging studies (MRI or 2DCT); (3) pB-C2 of 8.5 mm or above; and (4) dynamic basion dens interval (BDI) of 2.0 mm or more, as obtained during invasive cervical traction, and calculated by subtracting the BDI at end traction with the BDI off traction, both being measured in the sitting position.

### Radiological studies

**Fig. 1:**
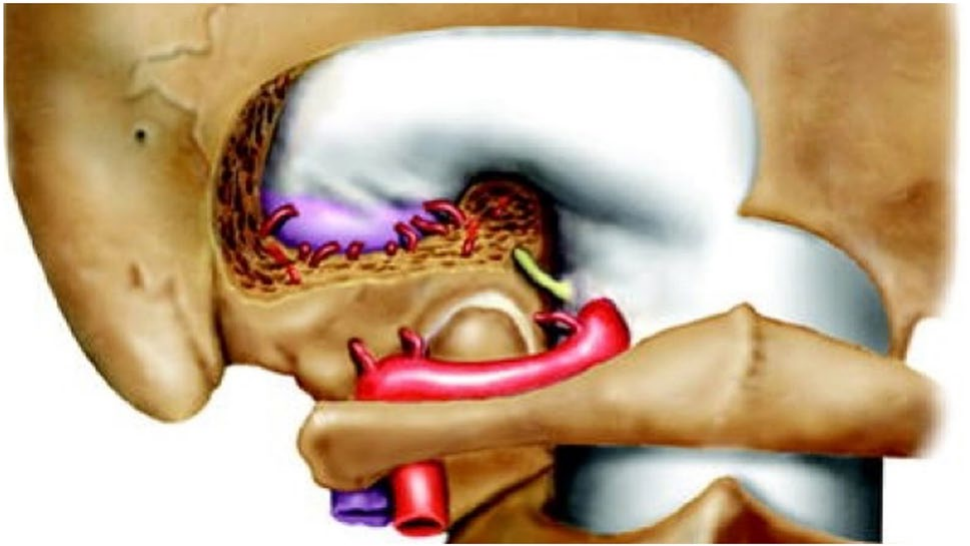
Sectional anatomy of occipital condyle

OsiriX**©** software was used on preoperative imaging to measure shape and dimension of the condylar anatomy, length and width of the bony corridors planned for screw insertion, and the extension of surgical bone defects (Fig. 1).

### Aspects of surgical technique

**Fig. 2:**
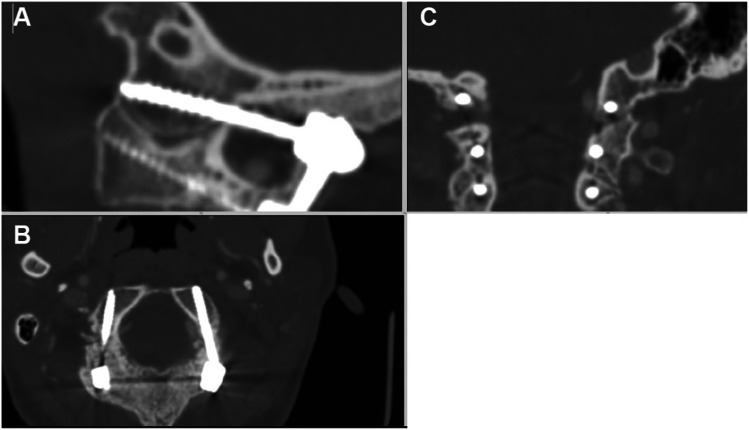
**A**–**C** Orthogonal 2D reconstructions (**A** sagittal, **B** axial, **C** coronal)

Over time, the entry point was shifted from the top to the middle third of the condyle, to avoid injury to the hypoglossal, especially with hypoplastic condyles. The sacrifice of the occipital emissary veins was key for an extensive dissection of the condyles, down to the C0-C1 space, which was violated to promote fusion within the joint. The hand drill was aimed around 10° medially, with variations dictated by the individual anatomy. The sagittal trajectory was aimed towards the basion under fluoroscopic guidance, with simultaneous EMG feedback from cranial nerve XII (Fig. 2).

Lag screws of 34 mm in length and 3.5 in diameter (Medtronic Vertex System) were inserted in the condyles, aiming to a bicortical purchase, with bone depths ranging from 18 to 22 mm. The insertion was followed by Doppler ultrasound examination of the vertebral arteries. Screw stimulation of the condylar screws was used after the first 25 cases, with a safety threshold assumed at 2.8 m [15].

In most cases, occipitocervical constructs comprised of condylar, C1 lateral mass, and C2 pedicle screws. The venous plexus at C1-2 was coagulated. The C2 ganglion was dissected and cut.

The condylar trajectories were checked with intraoperative CT scans. Two cobalt-chrome bars (3–4 cm long) were positioned in the screwheads bilaterally and locked in place with set screws. A slight bending and/or a side connector at C1 were required in some cases to obtain the best fit. A finely ground-up mix comprised of crushed cancellous allogenous cadaveric bone, autologous bone, Progenic gel, Mastergraft, and BMP-2 were laid over the hardware and the decorticated bone. BMP-2 was excluded in pediatric patients.

### Follow-up

All patients were scheduled for follow-up at 6 and 12 months postoperatively with MRI and CT imaging and then additional periodic follow-ups.

Of the 250 patients, 14 patients were lost to follow-up. One hundred twenty-five patients of the series have already passed the 5-year postoperative mark.

### Data acquisition and statistical analysis

The following data were tracked: (1) age and sex, (2) integrity of hypoglossal nerves, (3) integrity of vertebral arteries, (4) effects from the sacrifice of condylar veins, (5) hypoplastic condyles, (6) large suboccipital craniectomies, (7) congenitally fused C0-1, (8) post-surgical softening around the condylar screws, (9) post-surgical hardware breakdown, (10) post-surgical metal fatigue, (11) post-surgical painful profile, and (12) post-surgical occipital neuralgia.

Descriptive statistics were applied.

### Institutional Review Board approval

The retrospective record review pertinent to this study was approved by the Internal Review Board of the two Institutions where the surgeries took place. (IRB #13-655B: North Shore University Hospital – Northwell, Manhasset (NY) and WIRB #1,276,110: Mount Sinai South Nassau – Mount Sinai—Oceanside (NY)).

## Results

### Patient population

Two hundred fifty patients underwent OC fusion using a total of 500 condylar screws between September 2012 and September 2018. Two hundred seventeen patients were females. The mean age was 36 ± 13.4 SD, ranging from 6 to 75. 23 patients were below 18 years of age (ranging 6 to 17). Forty-four cases were revision surgeries from a former barplate configuration. Indications for revision operations included painful profile, metal fatigue, or bar-plate breakdown, alone or in combination. One hundred eighty patients (72%) had a formal diagnosis or clinical evidence of a connective tissue disorder. One hundred twenty-three patients (49.2%) had a suboccipital craniectomy to address Chiari I malformation as a comorbidity, thus satisfying the diagnostic criteria for Complex Chiari.

Complex Chiari was first described by Brockmeyer et al. and consists in a Chiari I malformation combined with an anatomical or functional disorder of the craniocervical junction [4]. The diagnostic prevalence of connective tissue disorder and Chiari I malformation in our series was an effect of the primary focus of our center.

### Skeletal issues

Hypoplastic condyles were identified in 91 out of the 250 patients (36.4%) (Table 1), but this finding did not prevent the condylar screw insertion in this series.

**Table.1:** Complications related to Condylar Screws and anatomical features affecting their placement

	Frequency	Percentage (%)
**Direct complications**		
Clinical compromise of the hypoglossal nerve	2	0.8
Clinical or hemodynamic effects of vertebral artery impingement	0	0
Venous strokes from the sacrifice of the condylar vein	0	0
Post-surgical softening around the condylar screws at the follow-up imaging	0	0
Post-surgical hardware breakdown	0	0
Post-surgical metal fatigue	0	0
Post-surgical painful profile	0	0
Post-surgical occipital neuralgia	0	0
**Anatomical features**		
Presence of hypoplastic condyles	91	36.4
Presence of congenitally fused C0-1	2	0.8
Presence of a large suboccipital craniectomy	5	2

Five cases had large suboccipital craniectomies extending up to the transverse sinuses and laterally to the sigmoid sinuses. In 97 out of the 123 patients with former Chiari decompressive surgery, a thickness of less than 2 mm residual available supraocciput was documented.

The occipital condyles and C1 were congenitally fused in two cases, and a single screw was inserted through these conjoined bony elements.

### Vascular issues

Condylar screws did not cause impingements or stenoses of the vertebral arteries in our study. There was no clinical evidence of stroke in the vertebral distribution attributable to the use of condylar screws. No clinical evidence of venous stroke was detected during the early postoperative period, or later in follow-up.

### Hardware issues

No condylar screw related painful profile was recorded in this study. Forty-four patients underwent a revision OC fusion from barplate configuration to condylar fixation. The new hardware was perceived to be more comfortable than the former.

Metal fatigue was not observed. No condylar screw pullout was detected at the 6-month follow-up and beyond. No hardware breakdown was found in this surgical series.

### Hypoglossal complications

Among the 500 condylar screws, two hypoglossal injuries were documented at the beginning of the series. Both cases involved patients with hypoplastic condyles and thin supraocciputs; the latter is considered as a limiting factor for the use of barplates. The first injury was caused by the screw impinging into the condylar canal. This deficit was not identified until after the operation as the neuromonitoring electrodes were mispositioned intraoperatively. A corrective surgery was performed promptly by screw repositioning through a new pass. A near complete recovery was observed at 6 months following the corrective operation. The second injury was attributed to hand drilling. The neuromonitoring promptly warned the surgeon, who redirected the drilling and the new screw trajectory. The postoperative deficit was moderate and the patient experienced a full recovery by the 6th postoperative month.

Corrective measures were adopted thereafter: (1) extending the dissection of the condyles, (2) lowering the entry point by 5 mm, and (3) shaving the overlaying supraocciput to allow a lower trajectory.

Three patients with hypoplastic condyles and screw trajectories clear of the hypoglossal canal had transient minimal unilateral tongue fasciculations, which resolved completely within 1–2 days, with no residual deficit.

### Complications not directly related to condylar screws

**Fig. 3:**
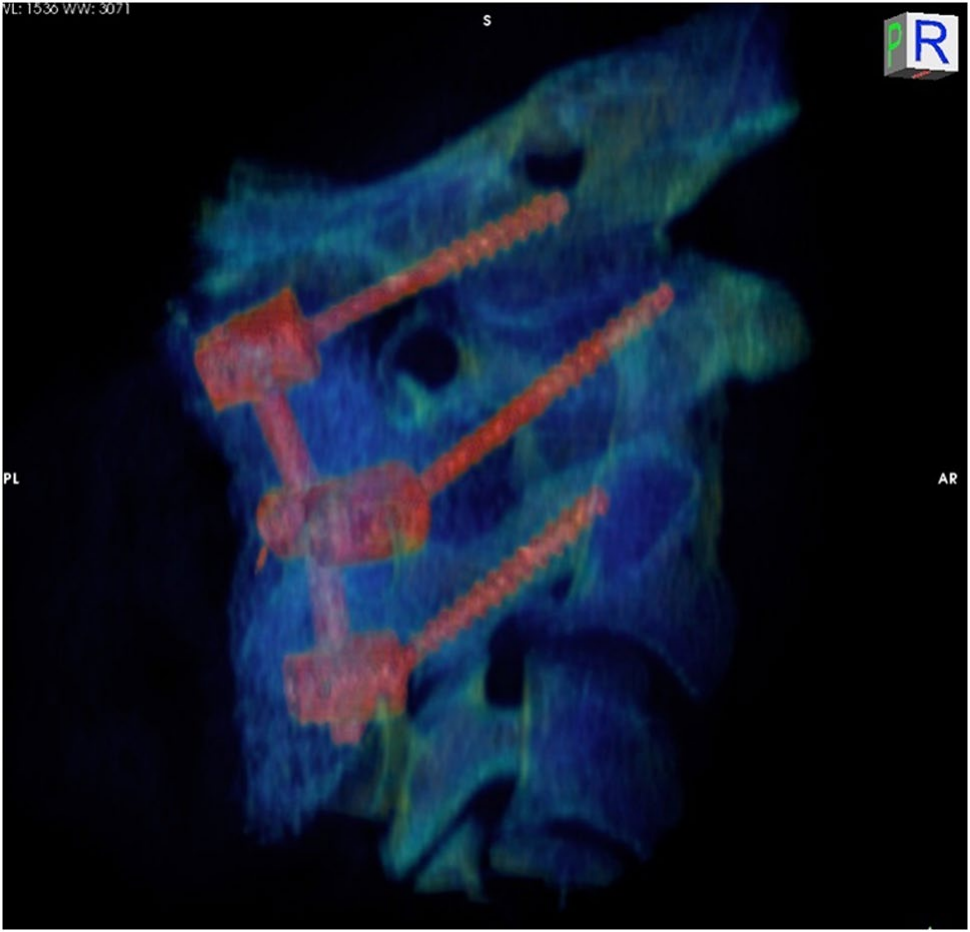
3D reconstruction of a C0-2 construct with condylar screws

Seven (2.8%) hardware revisions (Table 2), 25 (10%) wound revisions not requiring revision of the hardware (Table 3), and 20 (8%) extensions of the C0-C2 fusion to lower segments within the lower cervical of the thoracic spine were noted in our study (Fig. 3).

**Table.2:** List of cases requiring hardware revisions, not caused by condylar screw complications

• OC fusion revision, with osteotomies at C01-2 and repositioning in extension, to facilitate future endotracheal intubations
• OC revision, caused by an infected seroma, after a wound tap performed by a local MD
• OC fusion revision, after progression of rheumatoid arthritis, compromising the bone density at C1 and C2
• Broken C2 pedicle, with vertebral artery occlusion by the ipsilateral C2 pedicle screw
• OC revision of C2 screws (from pars to pedicle trajectories)
• Insertion of a crossbar in a former cranio-cervical-thoracic fusion
• Revision of the trajectory of two thoracic screws, in a former cranio-cervical-thoracic fusion

**Table.3:** Other complications not related to condylar screws

	Frequency	Percentage (%)
Wound revisions not requiring hardware revisions		
Subfascial pathologies		
Seromas	2	0.8
Hematomas	2	0.8
Infections	9	3.6
Section of a C2 nerve root post-amputation neuroma	1	0.4
Superficial wound dehiscence	11	4.4
Total	25	10
Caudal extension of the hardware		
Cervical	5	2
Thoracic	7	2.8
Cervical with subsequent thoracic	4	1.6
	16	6.4

### Fusion rate

At 12-month follow-up and beyond, the detected fusion rate was 100%.

## Discussion

Condylar screws have been introduced by Uribe et al. in 2008 as a novel rescue technique for OC fusions, in particular, in patients with history of craniectomy limiting the number and thickness of the available fixation points on the occipital squama [18]. Three trajectories were proposed, with different entry points medial to the condylar fossa to preserve the condylar vein [6, 12, 14, 17]. In this series, we did not have a fix trajectory, and we sacrificed the condylar veins to obtain a better exposure of the condyles.

The persistent interest in this technique has been reflected by a number of CT-based feasibility studies [13, 16, 19] and by recent surgical series which resulted in no complications [3, 10, 11].

A cadaveric feasibility study by Kirnaz et al. postulated that in 14.1% of adult OCs and 26.5% of pediatric OCs studied, placement of condylar screws would have been challenging or unsafe [9]. The anatomy of the condyles did not prevent the insertion of condylar screws in any of our series, despite the fact that hypoplastic condyles were identified in 91 out of the 250 patients (36.4%).

### Biomechanical considerations

**Fig. 4:**
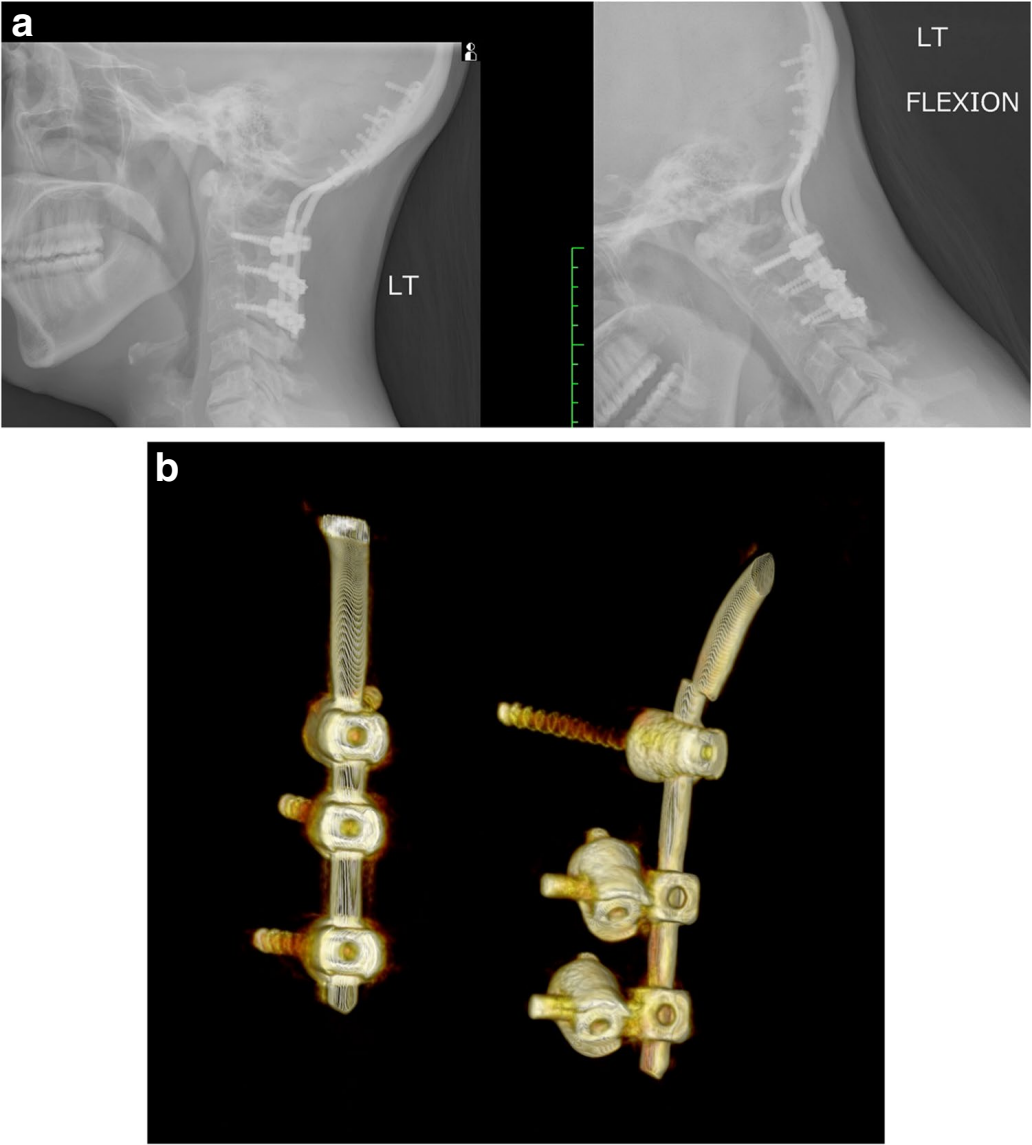
**A** X-rays cervical spine in flexion and extension. Lateral view of broken traditional barplate hardware. **B** 3D CT of broken traditional barplate, posterior view

**Fig. 5:**
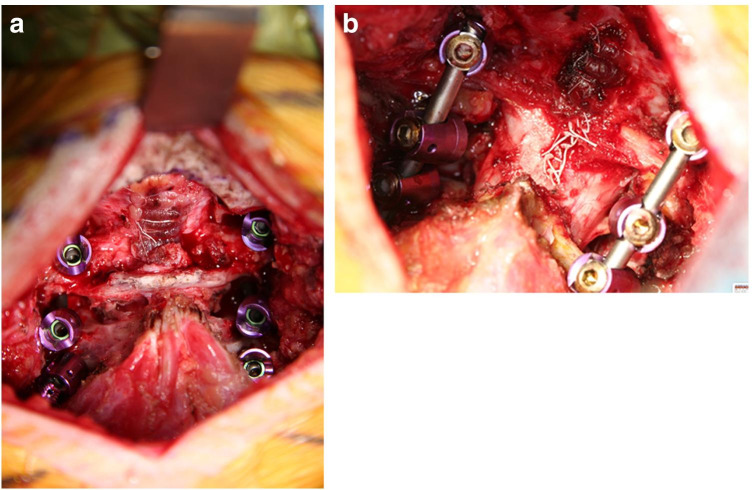
**A** Intraoperative photo of C0-2 construct with condylar screws, in a case with concurrent C1-sparing Chiari decompression, prior to the insertion of the bars. **B** Intraoperative photo of C0-2 construct with condylar screws, in a case with concurrent Chiari decompression, with C1 lamiectomy, after the insertion of the bars

**Fig. 6:**
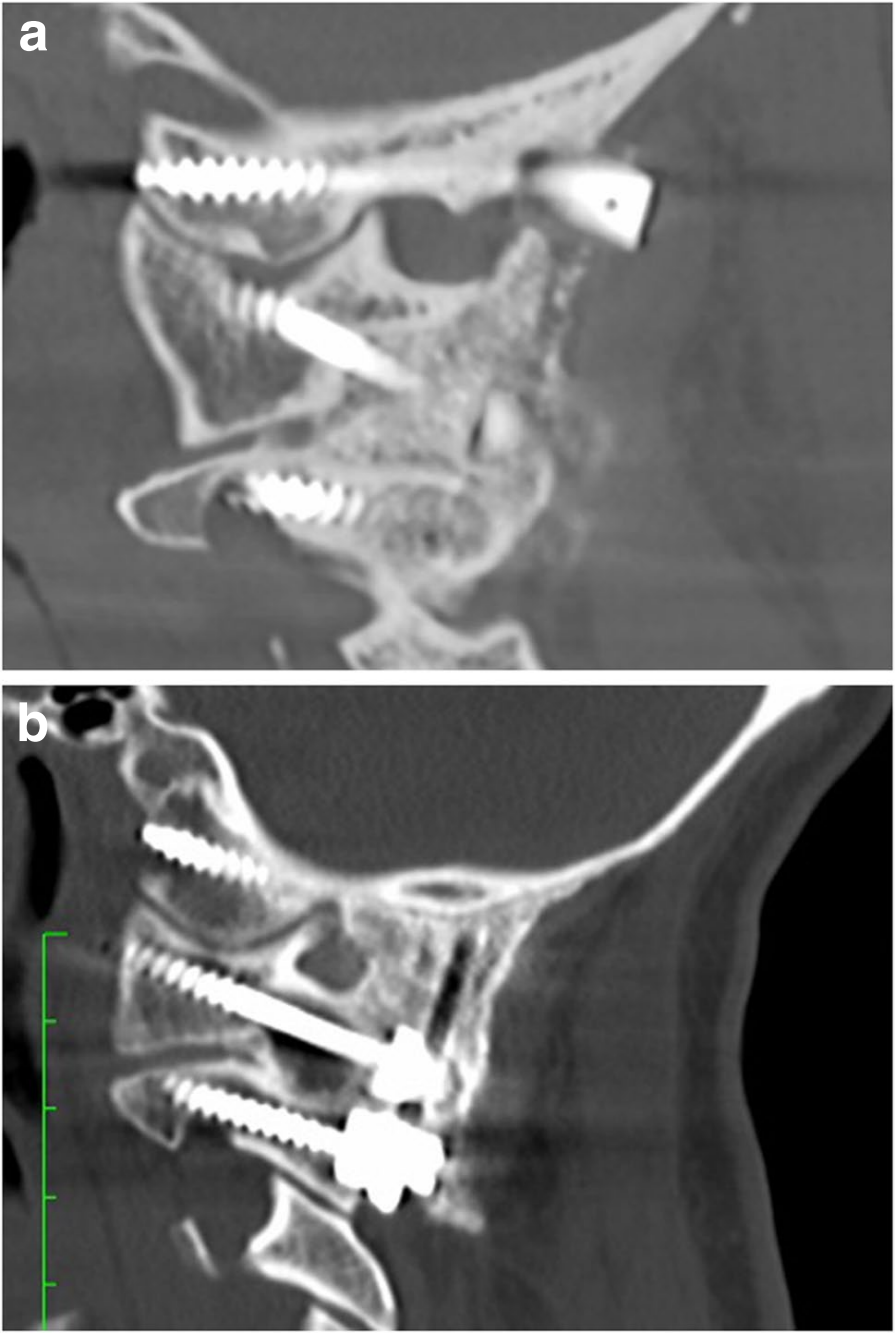
**A** Sagittal 2D CT showing condylar screw in hypoplastic condyle. **B** Sagittal 2D CT with C0-2 construct with condylar screws and extremely thin supraocciput

**Fig. 7:**
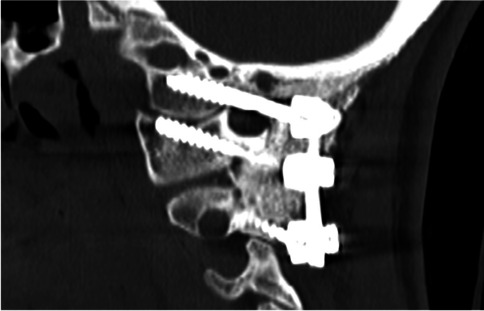
Sagittal 2D CT with C0-2 construct with condylar screws and mature fusion

Craniocervical stabilization using condylar and cervical screws has shown comparable results to standard barplate constructs, in terms of range of motion restriction and stiffness, in flexion, extension, lateral bending, and axial rotation (Fig. 4). Uribe et al. believe that decreasing the length of the lever arm of the construct, increasing the length of the screws with improved pullout strength, and use of the condyles as fixation points will provide for a more rigid OC construct [18]. Occipital condylar cervical fixation also covers less of the bony surface than barplates, allowing more surface area for osseous fusion and grafting (Figs. 5, 6, and 7).

Traditional barplates are L-shaped, curve at the craniocervical junction, and a have a gap between the upper cervical screw and the lowest supraoccipital screw. Metal fatigue tends to occur in the barplate within this curved segment, leading to recurrence of symptoms and in some cases hardware breakdown. The short length of the bar in the condylar construct, the close proximity of the screw heads, and the limited amount of exposed bar between the screw heads theoretically limit the risk of metal fatigue and breakdown. Within the constraints of the short follow-up, such complications were not observed in this surgical series to date.

When imaged with sagittal and coronal CT reconstructions, the anatomical arrangement of the condyles, the C1 lateral masses, the C2 lateral masses, and the screws arranged inside them closely resembles the postsurgical anatomy of an anterior cervical discectomy with fusion and instrumentation, thus suggesting a biomechanical comparison. In the case of long constructs extending from the occiput to the thoracic spine, it is our opinion that the condylar screw fixation should also be augmented by a barplate.

### Disadvantages and advantages of the technique

The position of condylar screws is in close proximity to important anatomical structures. The dissection of the condyle is challenging and requires a learning curve. Unlike other authors, we opted to sacrifice the condylar vein, to obtain the following: (1) an increased surface for the bone fusion, (2) an easier access to a wider condyle dissection, and (3) a decreased intraoperative blood loss, since an accidental laceration of a partially dissected condylar vein can be quite difficult to control.

Condylar screws do not require the placement of a screws through the occipital squama, thus avoiding the risk of CSF leakage, intraparenchymal hemorrhages, and the rare occurrence of transverse sinus injuries. Custom bending of the barplate to adapt to the profile of the bone anatomy is no longer required, and the problems caused by the hardware profile (pain, discomfort, muscle spasm, delayed wound healing) are greatly reduced.

The cobalt-chrome rods used in these constructs are short, require minimal or no bending, have a low profile, and allow for a wide fusion surface. Their configuration is likely to result in a long-term decreased incidence of metal fatigue and breakdown, when compared to standard barplate constructs. In addition, the exposure of the condyle allows placement of the graft material directly over the OC joint, thereby providing a graft site superior to that offered with onlay grafting. Despite former concerns, there were no accidental intraoperative lacerations of, or postoperative impingements on the Vertebral arteries, in this series.

Painful profile of the barplate component is a common complaint, linked to the rich local innervation by the greater occipitalis, and the thin layer of overlying soft tissue within the higher segments of the supraocciput.

Another long-term factor is the progressive soft tissue thinning caused by the profile of the barplate.

Unlike barplates, the condylar screw heads are surrounded by less innervated and thick cervical muscle, thus explaining why none of the patients followed up in this surgical series complained about painful profile referable to the condylar screws. In addition, all the 44 patients who underwent a revision of their original OC fusion from barplate configuration to condylar screw fixation stated that the latter hardware was more comfortable than the original one.

Metal fatigue was not observed in any of the 236 cases which reached follow-up, with 100 of these cases having already passed the 5-year postoperative mark. For the same reasons, no incidence of hardware breakdown was found in this surgical series. The main advantage of this construct versus the classic barplate configuration resides in the following factors: (1) the bar is short and mostly straight, (2) the vector of all the screws is pointing in the same direction, (3) a long portion of the bar is incorporated in the screwheads, and (4) the whole hardware is entirely encompassed by the mature bone fusion. To add anecdotal evidence, 3 patients of this series were involved in catastrophic high-energy motor vehicular accidents. The OC hardware remained intact in all three cases.

### Technical nuances

Compared to other authors, we have adopted a lower entry point on the condyle, and a flexible, non-fixed trajectory. This maneuver has allowed lower trajectories within the condyles, remaining clear of the hypoglossal canal even in the case of frequent occurrence of hypoplastic condyles. In the lateral projection, the basion is a reliable reference point to estimate the depth of the condylar screw insertion during fluoroscopic imaging.

The trajectory is mildly converging on the axial plane, with the horizontal pitch modified from case to case on the basis of the preoperative CT images.

The use of neuronavigation is recommended by a number of surgeons who have ventured with condylar screws [3, 10]. After using neuronavigation once, we instead relied on anatomical and fluoroscopic landmarks, which we found easy to master and reliable.

Intraoperative electrophysiologic monitoring is in our opinion a necessary component for the safe execution of this surgery. Moreover, its feedback information was instrumental in accelerating our learning process and in modifying and evolving our surgical technique [15].

### Hardware profile and induced discomfort

The length and the profile of these constructs compare favorably with barplates. In the years in which the main author used the standard barplates construct, several patients had received local block and trigger point injections, and 18 patients had neurostimulators implanted to deal with the local discomfort. None of the 236 patients seen in the follow-up had received similar forms of treatment to date. Another observed but hardly quantifiable by-product of the condylar series has been the decreased incidence of late postoperative muscle spasm in virgin fusion patients.

### Bone fusion

All the CT scans obtained post-operatively have shown solid bone fusions reinforcing the entire span of the hardware. The practice of violating the joint space at C0-1 has often resulted in solid fusions within these joint spaces. No BMP-2 associated complication was observed.

### Complications

Out of the 500 screw insertion in our series, only two complications were directly caused by condylar screws. In one case, a condylar screw encroached the hypoglossal nerve, while in the other the hypoglossal was involved during hand drilling. The corrective maneuvers, repositioning of the screw and redirection of the drilling, resulted respectively in a near complete and a total improvement of the deficits at the 6-month postoperative mark.

As a result of those adverse events, we soon adopted the following corrective strategies: (1) extending further the dissection of the soft tissues of the condyles, (2) shifting the entry point caudally, and (3) shaving the overlaying supraocciput, to allow a lower trajectory within the condyles.

Vertebral injuries, compressions, or dissections have not occurred in this case series as a direct consequence of the use of condylar screws. Venous infarction did not occur in this surgical series, despite the sacrifice of the occipital emissary vein. The emissary vein should probably be preserved in cases with pathological venous drainage patterns (i.e., Crouzon syndrome).

EDS and other connective disorders are characterized by poor wound healing. As a result of a higher prevalence of these diseases in our cohort, we adopted a lower threshold for superficial wound revisions to take place in an operating room.

### Limitations of this study

Despite the considerable size of this surgical series, the single-surgeon experience may limit the ability to generalize results. A larger series involving more surgeons and more centers would bear a stronger weight. The long-time biomechanical implications and possible delayed complications of the condylar screws of this series need to be further monitored before a definitive opinion can be formulated. There was a narrow spectrum of pathologies and genetic profiles involved in the study, and a relatively homogeneous sex and age distribution, reflecting the referral patterns of our quaternary center but did not represent the larger spectrum of the patients who can benefit from occipitocervical fusion in general. Since neuronavigation was not used in this series, our experience cannot point to the obvious advantages, or the possible shortcomings of this tool, when applied to this surgical technique.

## Conclusions

This surgical series suggests that the use of condylar screws fixation is a relatively safe and reliable option for OC fusion in both adult and pediatric patients. The incidence of complications recorded in this study was low, making this technique less technically challenging and less risky than initially anticipated. Methodical dissection of anatomical landmarks, intraoperative imaging, and neurophysiologic monitoring allowed the safe execution of the largest series of condylar screws reported to date. Separate contributions will follow in the future to provide details about the long-term clinical outcome of this series.
